# No evidence of uptake or propagation of reindeer CWD prions in environmentally exposed sheep

**DOI:** 10.1186/s13028-022-00632-3

**Published:** 2022-06-06

**Authors:** Erez Harpaz, Øyvind Salvesen, Geir Rune Rauset, Aqsa Mahmood, Linh Tran, Bjørnar Ytrehus, Sylvie Lafond Benestad, Michael Andreas Tranulis, Arild Espenes, Cecilie Ersdal

**Affiliations:** 1 Department of Production Animal Clinical Sciences, Faculty of Veterinary Medicine, Norwegian University of Life Sciences, Svebastadveien, 112, 4325 Sandnes, Norway; 2 Norwegian Institute for Nature Research (NINA), Torgarden, P.O. Box 5685, 7485 Trondheim, Norway; 3 Norwegian Veterinary Institute, P.O. box 64, 1431 Ås, Norway; 4 Department of Biomedical Science and Veterinary Public Health, Swedish University of Agricultural Sciences, P.O. Box 7028, 750 07 Uppsala, Sweden; 5 Department of Preclinical Sciences and Pathology, Faculty of Veterinary Medicine, Norwegian University of Life Sciences, Universitetstunet 3, 1433 Ås, Norway

**Keywords:** ELISA, Immunohistochemistry, Nordfjella, Norway, Prion, RAMALT

## Abstract

**Background:**

Chronic wasting disease (CWD) is a prion disease of cervids first reported in North America in the 1960s. In Europe, CWD was first diagnosed in 2016 in a wild reindeer in Norway. Detection of two more cases in the same mountain area led to the complete culling of this partially confined reindeer population of about 2400 animals. A total of 19 CWD positive animals were identified. The affected area is extensively used for the grazing of sheep during summers. There are many mineral licks intended for sheep in the area, but these have also been used by reindeer. This overlap in area use raised concerns for cross-species prion transmission between reindeer and sheep. In this study, we have used global positioning system (GPS) data from sheep and reindeer, including tracking one of the CWD positive reindeer, to investigate spatial and time-relevant overlaps between these two species. Since prions can accumulate in lymphoid follicles following oral uptake, samples of gut-associated lymphoid tissue (GALT) from 425 lambs and 78 adult sheep, which had grazed in the region during the relevant timeframe, were analyzed for the presence of prions. The recto-anal mucosa associated lymphoid tissue (RAMALT) from all the animals were examined by histology, immunohistochemistry (IHC) and enzyme-linked immunosorbent assay (ELISA), and the ileal Peyer's patch (IPP) from a subsample of 37 lambs were examined by histology and IHC, for the detection of prions.

**Results:**

GPS data showed an overlap in area use between the infected reindeer herd and the sheep. In addition, the GPS positions of an infected reindeer and some of the sampled sheep showed temporospatial overlap. No prions were detected in the GALT of the investigated sheep even though the mean lymphoid follicle number in RAMALT and IPP samples were high.

**Conclusion:**

The absence of prions in the GALT of sheep that have shared pasture with CWD-infected reindeer, may suggest that transmission of this novel CWD strain to sheep does not easily occur under the conditions found in these mountains. We document that the lymphoid follicle rich RAMALT could be a useful tool to screen for prions in sheep.

## Background

Chronic wasting disease (CWD) is a fatal neurodegenerative, contagious prion disease affecting members of the Cervidae family [1]. Like classical scrapie in sheep and goats, CWD spreads horizontally between individuals by direct contact or indirectly in the contaminated environment [2]. The causative agent for all prion diseases is a misfolded form (PrP^Sc^, or prions) of the host's normal cellular prion protein (PrP^C^). PrP^Sc^ accumulates in the central nervous system (CNS), and in some cases in peripheral organs and is the pathognomonic marker for prion disease [3, 4].

CWD was first described in mule deer (*Odocoileus hemionus*) in Colorado in the 1960s [5]. Since then, the disease has been discovered among free-ranging and farmed deer in at least 26 states in the USA and in three Canadian provinces, affecting white-tailed deer (*Odocoileus virginianus*), elk *(Cervus elaphus nelsoni*) [6], and occasionally moose (*Alces alces*) [7], in addition to mule deer. The disease has also been detected in South Korea, traced back to the importation of elk from Canada [8]. In 2016, CWD was diagnosed in a wild reindeer in the Nordfjella mountain region in Norway [9]. This was the first time the disease was found in Europe and was also the first natural case observed in reindeer.

**Fig. 1:**
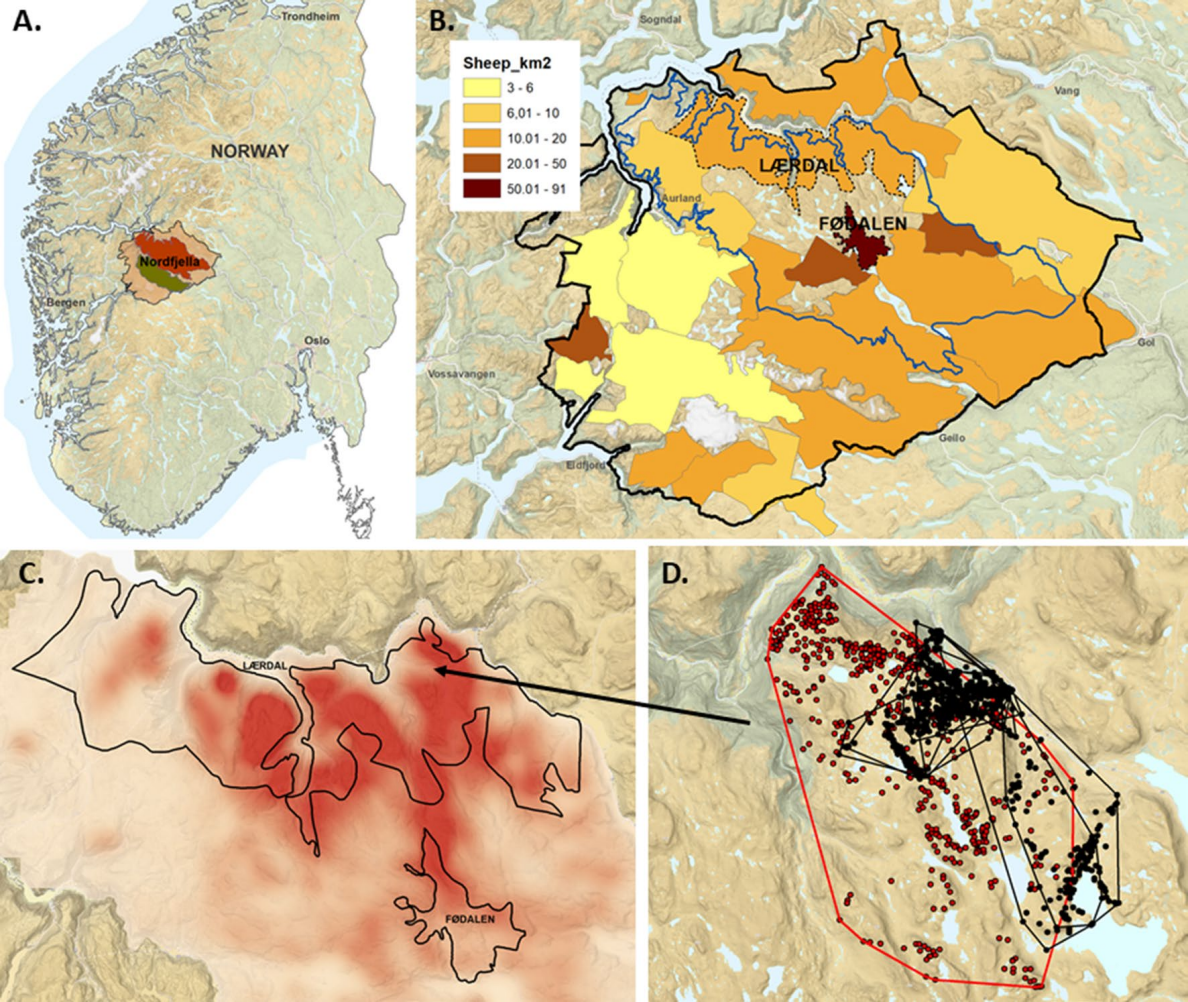
Nordfjella CWD management area, sheep and reindeer density and movements **A**. Location of Nordfjella CWD management area in Norway. Zone 1 is marked in red and zone 2 is marked in green. **B** Density of sheep released within the sheep grazing districts of Nordfjella in the spring of 2017. The zone 1 reindeer management area is outlined in blue. The sheep grazing districts Fødalen and Lærdal where the sampled sheep were grazing are marked with dotted black lines (Data source: Organisert beitebruk/NIBIO, https://kilden.nibio.no). **C** Estimated reindeer area use in Nordfjella zone 1 based on GPS locations from marked reindeer (26 females and 5 males) during 2007–2018. The more intense the red color, the more intense the area use. Sheep grazing districts Lærdal and Fødalen are marked with solid black lines. **D** GPS locations of adult female sheep (n = 5, corresponding to 10 individual grazing seasons) from the Lærdal grazing district included in the study (black dots and lines) and a CWD-positive reindeer that was marked on the 29th of March and culled the 21st of June 2017 (red dots and lines) showing overlapping area use. The dots in this figure represent individual positions of the animals, while the lines show the borders of the total area that was used by an individual animal per season

Nordfjella is a mountainous area in South Norway (Fig. 1A), which was defined by the authorities as “management area” following the CWD outbreak. Within this area there are two important subareas: zone 1, the area of the CWD outbreak, and zone 2, in which a smaller reindeer herd is still present (Fig. 1A). Since the affected population in zone 1 was partly confined, the population was removed from the area by combined hunting and culling during autumn 2017 and winter 2018 to prevent the spread of the disease to zone 2 and further spread to, not only the adjacent wild reindeer population on the Hardanger mountain plateau, but also to semi-domesticated reindeer in the north, and other cervid species in the surrounding valleys. All reindeer from zone 1 were tested for CWD and 19 of the 2424 animals tested were positive (0.8%). The mean age of the affected reindeer was 3.8 years [10].

Subsequent to the initial identification of contagious CWD in reindeer, CWD has also been observed in moose in Norway (n = 9, all were 12 years or older), Finland (n = 2) and Sweden (n = 4) (for all Nordic cases the mean age was 14,5 years), in addition to three cases in red deer (*Cervus elaphus*) in Norway. It has not been possible to establish any epidemiological connections between the outbreak in reindeer and the cases in moose and red deer [11]. Further investigations, including prion strain characterization in bioassays, have established that the red deer and moose cases represent prions strains that are different from each other. They also differ from Norwegian reindeer CWD and from CWD strains in North America [12, 13]. They are thought to represent sporadic prion disease as has been described in other species, rather than contagious CWD [14–16]. In 2020, a new case of contagious CWD was discovered in a wild reindeer on the Hardanger mountain plateau south of Nordfjella [17], representing a new geographical region for infection. As of April 2022, no additional cases have been found amongst the 5547 reindeer tested from this region [18].

**Fig. 2:**
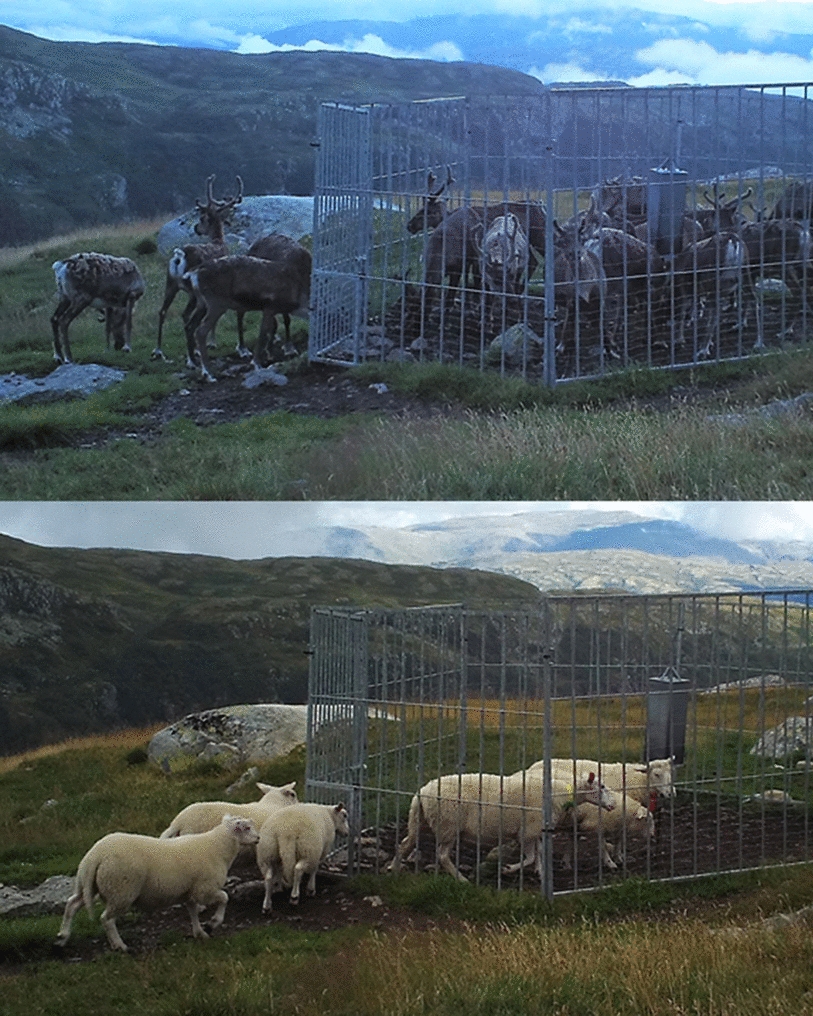
Sheep and reindeer visiting the same mineral lick site in Nordfjella zone 2 in 2018. The two pictures were taken by a camera trap four hours apart. At the mineral lick sites, the animals lick the salt stone and other surfaces, as well as ingesting soil, and grazing. They are consequently exposed to saliva, urine, and feces from previous visitors. The fences were supposed to keep reindeer and other cervids outside the salt lick site, while allowing sheep to crawl through the 40 cm × 53, 5 cm openings. This did not work as planned and the openings were subsequently closed later that same year

Nordfjella is a high-quality grazing area for sheep. It is divided into smaller grazing districts, which are used by both local and distant farms, with hundreds of permanent mineral licks established throughout the area [19]. During the summer of 2017, approximately 70,000 sheep were released into the area and 52,000 of these grazed in zone 1. The density of sheep within the zone varies, with some grazing districts such as Fødalen having a density close to 100 sheep/km^2^, while other districts as Lærdal has a lower sheep density of around 20 sheep/km^2^ [19] (Fig. 1B). Data from on-going research in Nordfjella and other regions show that mineral licks also attract reindeer and other cervids, not just sheep, leading to considerable indirect interactions between these species (Fig. 2). After the detection of CWD, some of the mineral licks were fenced off and closed permanently, whereas the remaining mineral licks were fenced, but equipped with openings that were thought to allow sheep to pass, but not cervids. This did not work as planned, since both reindeer and red deer were able to crawl through the openings and gain access to the mineral licks [19], and the openings were eventually closed. It is likely that both mineral licks [20] and water [21] can serve as environmental reservoirs for CWD prions and facilitate their spread and transmission potential.

The close interaction between reindeer and sheep in Nordfjella has raised concerns regarding potential interspecies transmission of CWD, an event that would have far-reaching social and economic implications. The possibility of propagation of CWD prions in sheep, which return to home farms and could spread the disease, would be exceedingly challenging to discover and control. It is therefore important to identify and characterize the degree of contact and overlap between sheep and reindeer in zone 1 in Nordfjella, and to test sheep that have grazed in the area for prion uptake and accumulation.

Definite diagnosis of prion disease in animals is based on the detection of prions in the CNS or lymphoid tissue by ELISA, immunohistochemistry and/or Western blot tests [22]. In early subclinical cases of scrapie or BSE in sheep, prions in the brain are sparse or not detectable [23–25], while there can be a significant accumulation of prions in peripheral lymphoid tissues [26, 27]. In sheep with scrapie [28, 29] and cervids with CWD [30, 31] oral exposure leads to uptake and early accumulation of prions in the gut associated lymphoid tissue (GALT), draining lymph nodes and the enteric nervous system (ENS). In naturally infected and highly susceptible lambs, scrapie prions have been detected in the ileal Peyer’s patches (IPP) and the mesenteric lymph nodes as early as two months of age [32]. Another study of natural scrapie infection described accumulation of prions in the IPP of three-month-old lambs harboring other, less susceptible prion protein (PrP) genotypes [25]. Thus, early detection of prions in subclinical animals is possible through the examination of lymphoid tissues. Lymphoid tissues which are available to sample via minimally invasive procedures include the 3rd eyelid [33], tonsils [34], and the recto-anal mucosa associated lymphoid tissue (RAMALT) [35, 36]. RAMALT is found just proximal to the mucocutaneous border of the anal opening and is rich in lymphoid follicles [37]. Its distal and easily accessible location makes it an attractive sampling area for the diagnosis of prion disease both in live animals [38, 39] as well as at post-mortem [35].

This study aims to investigate environmental exposure to prions, with possible uptake and aggregation in lymphoid tissues, in sheep that have grazed in the areas used by the CWD affected reindeer population in Nordfjella, Norway. Firstly, we investigated overlaps in area usage between sheep and reindeer before examining GALT (RAMALT and IPP) from slaughtered sheep from two grazing districts for prions. We hypothesized that although there is considerable overlap in area use and multiple meeting points with potential for transmission, CWD prions are not transmitted from reindeer to sheep.

## Methods

### Animals

The majority of the material sampled was from sheep that grazed in Fødalen grazing district where approximately 3200 sheep were on summer pasture in 2018. Out of these, 475 animals (~ 15%) were sampled during slaughtering at the Nortura Gol abattoir on September 2^nd^, 2018. Based on data available from the slaughter list and ear tags, 45 animals (9.4%) were adults, with the oldest being 8 years old and the remaining 430 animals being lambs. All lambs had grazed one summer (typically mid-June to early September) in Fødalen valley in Nordfjella zone 1 (Fig. 1B), while most of the adult sheep had most likely grazed more than one summer in the region. In addition, nine adult sheep from the Lærdal sheep grazing district were sampled at the Nortura Førde abattoir in March 2020, and a further 19 adult sheep were sampled in October 2020. These adult sheep had grazed Nordfjella zone 1 for one summer or more while reindeer were still present (Fig. 1B). A summary of the number of sampled animals and age groups is presented in Table 1. The management of the abattoirs and the farmers had given their consent to perform the sampling.

**Table 1:** Age distribution of the sampled sheep from Nordfjella at the time of slaughter

Age in years	N	Percentage of total
0.5	430	85.5
1–3	23	4.5
4–6	30	6
> 6	20	4
Total	503	100%

### Sampling procedure

Immediately following slaughter, the terminal part of rectum was removed, by making a circular incision around the anus as with standard slaughtering procedures. The follicle-rich lymphoid tissue, RAMALT, was sampled by cutting a rectangular area of mucosa from the mucocutaneous junction of the rectum and extending the cut approximately 2 cm cranially with a scalpel [36]. The RAMALT tissue samples were then divided in half horizontally. One half was fixed in 4% formaldehyde solution, and the second half was placed in an individually labelled plastic bag and frozen at − 70 °C until analysis. In addition, a section of the ileum, containing IPP, was taken from 37 of the lambs from the Fødalen grazing district and fixed in 4% formaldehyde solution.

### Histology

The formaldehyde fixed samples were prepared for sectioning by orientating them perpendicular to the surface in a longitudinal direction prior to dehydration in graded ethanol and embedding in paraffin. Three µm sections were taken, transferred to glass slides, deparaffinized, and stained with hematoxylin and eosin for histological analysis. The number of lymphoid follicles were counted on low magnification. Samples with six or more lymphoid follicles were categorized as “sufficient”, while those with less than six lymphoid follicles were categorized as “insufficient” [40]. In rectum sections, the presence or absence of cutaneous stratified squamous epithelium (SSE) was also noted as an indicator for the location of sampling with relation to the recto-anal junction [38]. If present, the amount of SSE was judged subjectively. The scoring for SSE was as follows: 0, no SSE present; 1, < 50% of the section was covered with SSE; 2, > 50% of the section was covered with SSE.

### Immunohistochemistry

Paraffin-embedded samples were sectioned at three µm and placed on positively charged glass slides (Superfrost Plus^®^, Menzel-Gläser, Thermo Scientific, Oslo, Norway). The sections were deparaffinized with xylene and graded ethanol in decreasing concentrations and then immersed in 98% formic acid for 15 min prior to incubation in a citrate buffer bath (pH 6) for 15 min at 121 °C. Following cooling, endogenous peroxidase activity was inhibited by 3% H_2_O_2_ diluted in methanol for 15 min, and non-specific binding sites were blocked with 1:50 goat N-serum in 5% bovine serum albumin (BSA) for 20 min. The samples were then incubated with the primary PrP antibody F99/97.6.1 (VMRD, Pullman, Washington, USA) diluted 1:800 with BSA 1% for 60 min at room temperature. The remaining procedure was performed using a commercial kit (Envison + K4005, Agilent Dako, California, USA). The secondary antibody conjugated to horseradish peroxidase (HRP) was applied for 30 min, and signals were visualized by incubating the slides in 3-amino-9-ethylcarbazole (AEC) for 10 min. Counterstaining was performed with hematoxylin for 1 min. Each run included one scrapie-positive control and one negative control, in addition to a methodological control in which the primary antibody was substituted with 1% BSA.

### Enzyme-linked immunosorbent assay (ELISA)

All RAMALT tissues were stored at − 70 °C prior to analysis. ELISA analysis was performed using a commercial kit validated for scrapie and BSE detection in the brain, spleen, and lymphoid tissue (HerdChek* Bovine Spongiform Encephalopathy-Scrapie Antigen Test Kit, IDEXX Laboratories, Inc., Westbrook, ME, USA). Approximately 300 mg of RAMALT tissue was cut into small pieces and placed in the grinding tube provided in the test kit for tissue homogenization. The samples were then subjected to 4 cycles of 30 s each at 6.5 m/s using a ribolyser (FastPrep fp120 cell disruptor; Thermo Savant, USA). The samples were allowed to cool for 5 min between each cycle. The remaining procedure was performed according to the manufacturer’s instructions. Briefly, 100 µL of the sample homogenate was added to a dilution plate well along with 50 µL diluent and mixed gently. A 100 µL subsample from each dilution plate well was pipetted into wells on the antigen capture plate and incubated at room temperature for 2 h. The wells were washed seven times with the wash solution provided in the kit. The conditioner buffer was then added to each well and the plate was incubated for 10 min at room temperature. After washing the wells three more times, the conjugate was added, and the plate incubated for 60 min at room temperature. The wells were then washed five times. After the addition of 100 µL substrate, the plate was incubated for 15 min at room temperature. Finally, an acid stop solution was added, and the optical density of the samples was read at 450 nm and 620 nm using an automated 96-well spectrophotometer (Thermo Scientific Multiskan go, ThermoFisher, USA). The cut-off values were calculated by adding 0.180 to the mean optical density of the two negative controls in each plate. Samples with a value above the calculated cut-off were deemed positive, while samples with a value under the cut-off were negative.

### Overlap in reindeer and sheep area usage

Reindeer location data was collected in total from 29 reindeer (24 females, five males) outfitted with global positioning system (GPS) collars (Vectronics GPS + and GPS PRO LIGHT) at various time points from 2007 until the Nordfjella zone 1 herd was culled in 2018. The GPS collars were put on in the winter after chemical immobilization, following standard procedures at the Norwegian Institute for Nature Research approved by the Norwegian Animal Research Authority (application identity numbers (FOTS id) 2375, 3993, 6052 and 8558). The GPS data was recorded as one location every three hours.

The intensity of reindeer area use was estimated using fixed kernel utility distribution (UD) with a bivariate normal kernel and 0.4*h as the smoothing parameter, based on the adehabitatHR library [41] in R (https://www.R-project.org/). The smoothing parameter was selected using a method proposed by Kie [42], sequentially reducing the reference bandwidth ‘h’ with 0.1 increments (0.9*h, 0.8*h, etc.) until balancing a close fit to original GPS locations and avoiding fragmentation.

Sheep location data from five of the abattoir sampled ewes belonging to the Lærdal grazing district and outfitted with GPS collars (Findmy Model1) during the summer seasons 2016–2020 were included in the study. These data corresponded to 10 individual grazing seasons as some of the sheep carried the GPS collar for more than one season. To evaluate overlap in area usage between these sheep and a GPS collared reindeer with verified CWD (data collected in the period 29 March 2017–21 June 2017), individual seasonal home ranges were estimated using 100% Minimum Convex Polygons (MCPs). The use of mineral lick sites by sheep and wildlife was monitored using motion-triggered camera traps (Recognyx XP9 UltraFire and Browning Strike Force HD).

### Statistical analysis

For statistical analysis of histological findings in RAMALT and IPP slides, a one-way ANOVA with Tukey post hoc test and t-test were performed using GraphPad Prism version 6.07 for Windows (GraphPad Software, San Diego, California USA, www.graphpad.com). Microsoft Excel (v.16.0.13801.20442) was used for descriptive statistics.

## Results

### Histology and immunohistochemistry

**Fig. 3:**
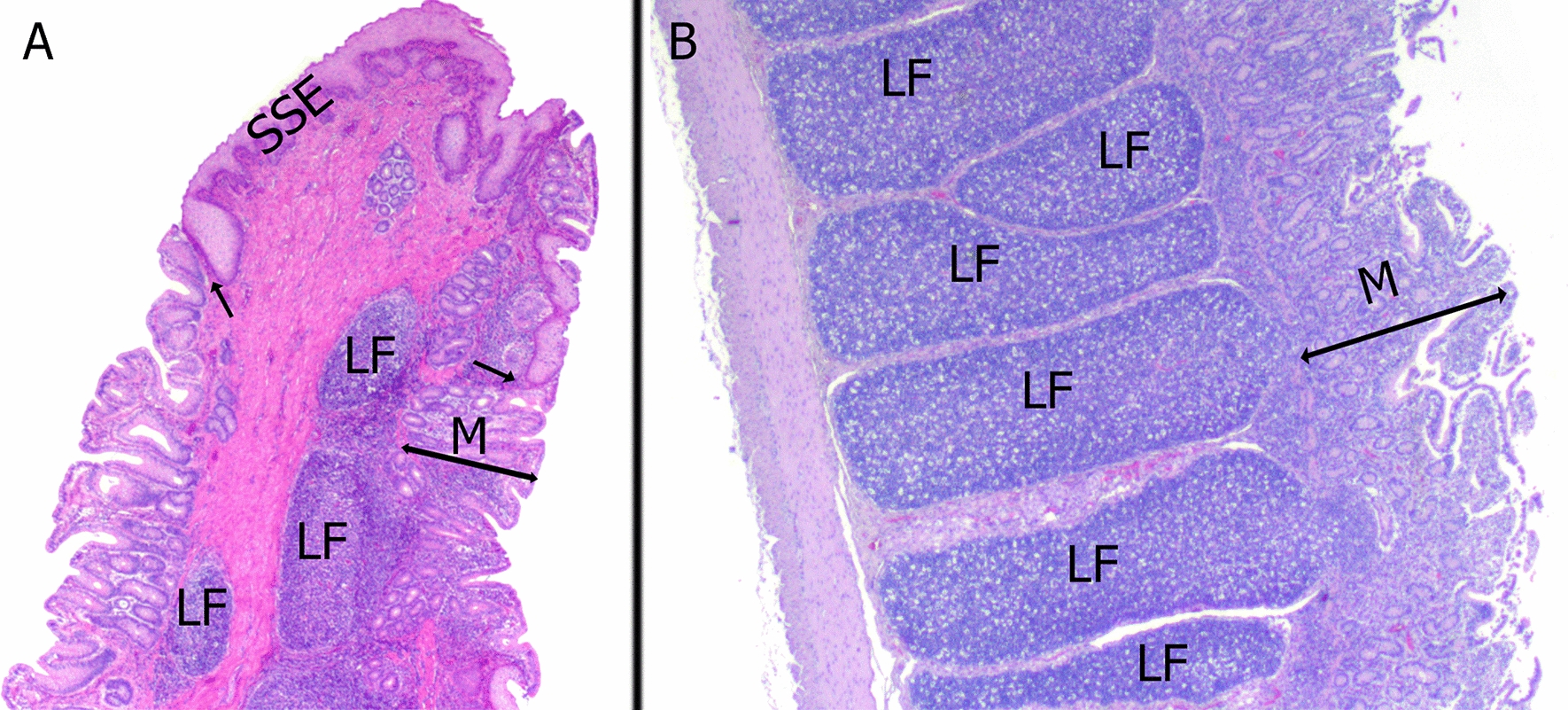
Representative histological sections of sampled gut-associated lymphoid tissue from sheep sampled at slaughter from the nordfjella region, norway. **A** Sheep rectum: a RAMALT section that contains less than 50% stratified squamous epithelium (SSE). Lymphoid follicles (LF) are found next to the mucocutaneous junction (arrows) **B**. Sheep ileum (IPP): the lymphoid follicles (LF) in the ileal Peyer’s patch are elongated and more densely packed in comparison to the RAMALT. M—mucosa. Hematoxylin and eosin, magnification 250x

In total, 503 RAMALT sections comprising 12,746 lymphoid follicles were analyzed (Fig. 3A). A minority of the sections came from adult sheep that had grazed one or more seasons in Nordfjella (14.5% of all samples) and the remaining were from lambs (85.5%) that had only grazed for one season. All lambs had grazed in Fødalen while adult sheep came from either Lærdal or Fødalen sheep grazing districts (Fig. 1B). None of the histological sections, when stained with the F99/97.6.1 antibody showed immunolabeling indicative of the presence of prions.

**Fig. 4:**
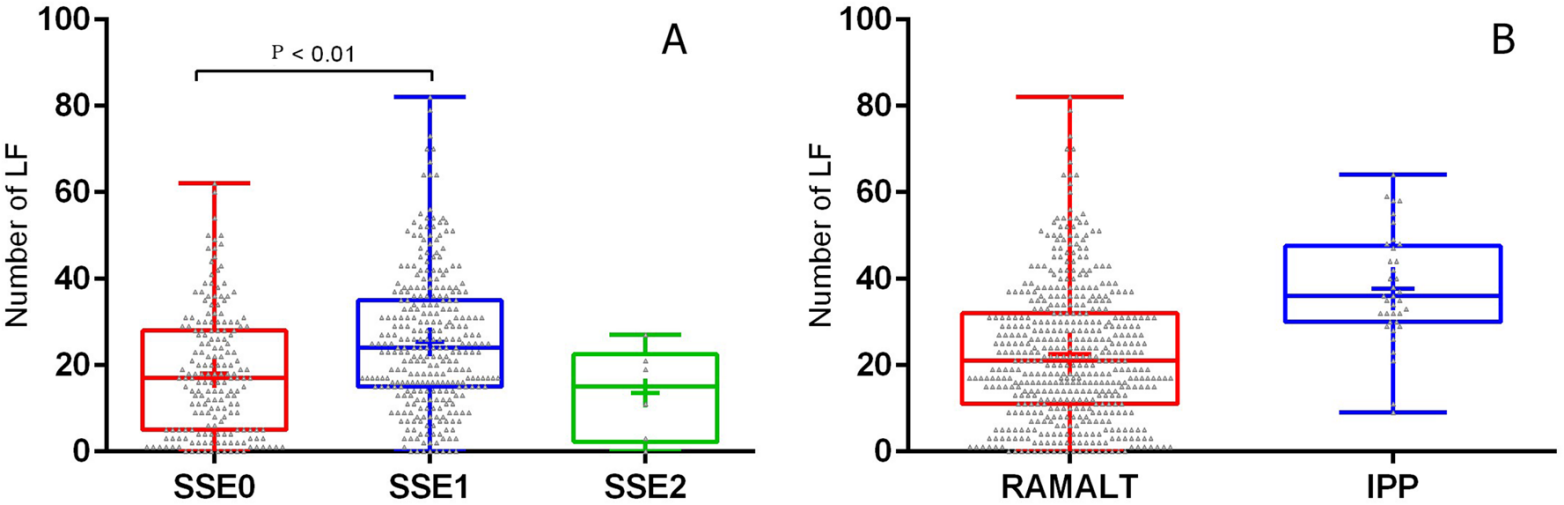
Distribution of lymphoid follicles in the RAMALT and IPP in sheep **A**. Distribution of lymphoid follicles in the different scoring groups based on the amount of stratified squamous epithelium (SSE) present in RAMALT sections. The number of follicles is significantly different between SSE0 and SSE1. **B** Distribution of lymphoid follicles in the RAMALT and IPP. SSE0 – No SSE present. SSE1—< 50% SSE. SSE2—> 50% SSE. Whiskers represent the minimum and maximum number of lymphoid follicles. The box represents the 2nd and 3rd quartiles. The middle bar is median, + is mean. The dots represent individual samples

The mean number of lymphoid follicles per section of RAMALT was 22.6 (95% CI 21.2–23.9). However, 16.3% (82/503) of the sections examined had less than six follicles (insufficient) and 3.7% (19/503) contained no follicles (Table 2). All the IPP sections were rich in lymphoid follicles (Fig. 3B) with the mean number being 37.8 (95% CI 33.5–42). The number of lymphoid follicles per section was significantly higher in IPP compared with RAMALT (P < 0.01) (Fig. 4).

**Table 2:** Comparison of the number of lymphoid follicles (LF) between rectum (RAMALT) and ileal Peyer’s patch (IPP) in sheep from the Nordfjella region in Norway, sampled at slaughter in 2018 and 2020

Sample type	n	Mean LF	95% CI	Median LF	Samples containing < 6 LF (%)	Samples without LF (%)
RAMALT	503	22.6	21.2–23.9	21	82 (16.3%)	19 (3.7%)
IPP	37	37.5	33.5–42	36	0	0

*n* number of samples

The total area of SSE covering the section was subjectively estimated as being above or below 50%. The majority of the Sects. (308/503) contained SSE which covered less than 50% of the tissue (SSE1) (Fig. 3A), and the mean number of follicles in this material was 25.5 (95% CI 23.7–27.2). There were significant differences between the follicle counts in samples with less than 50% SSE coverage and those without SSE coverage (mean follicle count 18.1; 95% CI 16.1–20.2), but no significant differences with samples with more than 50% SSE coverage (mean follicle count 13.5; 95% CI 2.3–24.7), (Table 3; Fig. 4).

**Table 3:** Number of lymphoid follicles (LF) and amount of stratified squamous epithelium (SSE) in RAMALT sections in sheep from the Nordfjella region in Norway, sampled at slaughter in 2018 and 2020

	n	Mean LF	95% CI	Median LF	Number of samples with < 6 LF (%)
SSE 0	189	18.1	16.1–20.2	17	52 (27.5%)
SSE 1	308	25.5	23.7–27.2	24	28 (9%)
SSE 2	6	13.5	2.3–24.7	15	2 (33%)
Totals	503	22.6		21	82 (16.3%)

n number of samples. SSE 0—No SSE present. SSE 1—< 50% SSE. SSE 2—> 50% SSE

### ELISA

PrP^Sc^ ELISA was performed on 515 RAMALT samples bordering the histological samples, 12 more than were analyzed histologically and by immunohistochemistry. The identifying number on 12 of the histological samples was not legible and these samples were therefore excluded from histological analysis, resulting in this discrepancy. The values of all samples analyzed by PrP^Sc^ ELISA were lower than the estimated cutoff value and were therefore considered to be negative.

### Overlap in reindeer and sheep area usage

We identified area usage overlaps between reindeer and sheep at all the spatial scales investigated. There was historical area usage by the diseased reindeer population in both the Lærdal and Fødalen sheep grazing districts. There was overlap between the area covered by a CWD infected reindeer individual that was GPS tracked during spring 2017 and the five GPS tracked ewes during summer grazing periods 2016–2020. Lastly, there was spatiotemporal overlap in the utilization of local resources (Figs. 1B–D). The two sheep grazing districts that were sampled had relatively high to very high densities of sheep (Fig. 1B) and were districts that had been extensively used by reindeer before they were culled (Fig. 1C).

Sheep and reindeer both visited the highly attractive mineral lick sites and licked or gnawed on soil and other surfaces (Fig. 2). Consequently, they were exposed to saliva, urine, feces, and other excretions from previous visitors to the lick sites, potentially also from infected reindeer shedding prions.

## Discussion

The present study was performed in response to the recent outbreak of CWD amongst reindeer in Norway. The outbreak raised concerns about the potential spread of CWD to wildlife and to the high number of sheep that graze in the region of the affected reindeer population. This concern still exists despite the depopulation of the reindeer in Nordfjella zone 1 [19] since prions have been shown to maintain infectivity in the environment for a long time [43–45].

The potential transmission of CWD from reindeer to sheep will depend on the degree of habitat overlap between the two species. Given that such overlap exists, factors like the amount of prions available in the environment [46], to which degree sheep ingest contaminated material (by licking contaminated surfaces or ingesting contaminated food, soil, or water) and the ability of prions to infect and propagate in new host species [20, 32], are important. Studies of several cervid species in North America have shown that prions are present in peripheral tissues and shed in saliva and urine [47], feces [48], and antler velvet [49]. A similar tissue distribution of prions was found in the Norwegian reindeer cases [9], suggesting that shedding of such infectious material also occurs with these animals. Environmental contamination of prions will depend on the number of infected animals, but other factors such as climate and soil characteristics [50–52] will impact the availability and persistence of prions in the environment. The high degree of overlapping area use between the affected reindeer herd and the sampled sheep populations (Fig. 1) and the presence of numerous mineral lick sites (Fig. 2), which have been regarded as high-risk spots for environmental prion contamination and potential cross-species transmission [20, 53, 54], indicate that some of the sampled sheep probably have been exposed to CWD prions.

In the present study we sought to find evidence of a reindeer to sheep transmission, under natural conditions. Such evidence was not obtained with the methods applied, despite investigating more than 500 sheep that had grazed in areas also used by the infected reindeer population. Around 85% of the available sheep examined for prion uptake were lambs born during March/April and sampled in September the same year. Generally, oral uptake of prions and further propagation of the disease is more efficient in young lambs compared to adult sheep [55, 56]. But as we don’t know enough about the kinetics of prion aggregation during infection with Norwegian reindeer CWD in sheep, the sensitivity of using lambs as indicators of trans-species transmission is difficult to assess. The older sheep that had grazed in the same area for several seasons could be better for demonstrating propagation and aggregation of prions in the tissues, given that infected reindeer were still present in the area concurrently with these animals’ first grazing season on pasture. Sheep of all age groups investigated in the present study were included due to their history of close temporospatial contact with the infected reindeer population and because tissues from these animals were accessible at the abattoir.

The transmissibility of various CWD-isolates to sheep has been investigated in several models [57, 58]. A recent paper showed subclinical peripheral prion infection in one out of seven sheep, 60 months after oronasal inoculation with a low dose of brain material (0.1 g) from a white-tailed deer with CWD. Prions were found by IHC in the retropharyngeal lymph node and palatine tonsil of an ARQ/ARQ sheep, harboring one of the most common PrP-genotypes, whereas no prions were found in two VRQ/ARQ sheep, harboring a PrP-genotype more susceptible to classical scrapie [57]. The range of investigated tissues was limited and did not include RAMALT or other parts of GALT, that are still present at this age as jejunal Peyer’s patches or lymphoid patches in the colon. A study using intracerebral inoculation of North American mule deer CWD prions into eight 4-month-old Suffolk lambs reported that only the one sheep with the PrP-genotype VRQ/ARQ developed clinical disease at 35 months post inoculation (MPI) [58], although one of the sheep with the ARQ/ARQ PrP-genotype was subclinically infected upon euthanasia at 72 MPI. Two other ARQ/ARQ and four ARQ/ARR inoculated sheep did not accumulate prions [58]. The prions were able to adapt to the new host and increase the attack rate after a second intracranial passage of the disease in sheep [59]. In a conference paper, sheep were reported to be infected when challenged intracerebrally with elk CWD, but not after oral inoculation [60]. The seemingly strong species barrier between cervids and sheep was also indicated by intracerebral inoculations of transgenic mice expressing sheep PrP (ovinized) with white-tailed deer CWD and transgenic mice expressing cervid PrP (cervidized) with classical scrapie, both of which showed very low attack rates [61]. However, it is important to acknowledge that transmission studies using North American CWD isolates must be interpreted with caution. Several recent papers report differences between Norwegian and North American CWD isolates [13, 62, 63]. Norwegian reindeer CWD has a remarkably low attack rate in bank voles [12] but transmits efficiently into transgenic and gene-targeted cervidized mice, albeit with longer incubation times than North American moose CWD [62]. An in vitro protein misfolding cyclic amplification (PMCA) study suggests that Norwegian reindeer CWD may be transmitted easier than North American isolates to sheep, cattle, hamsters, and mice (all transgenic mice substrates), but less efficiently to humans (substrate from humanized mice) compared to North American isolates [63].

These apparent differences need to be investigated further by a range of models, including experimental inoculations of Norwegian reindeer CWD in sheep. Thus, based on the currently available studies, is difficult to speculate how different sheep PrP genotypes might influence the ovine susceptibility to Norwegian reindeer CWD. As a part of the Norwegian surveillance program of scrapie in small ruminants a representative subsample of the investigated sheep was yearly PrP genotyped up to 2016 [64]. The prevalence of the most susceptible PrP genotypes towards classical scrapie (VRQ/VRQ, VRQ/ARQ, VRQ/ARH, VRQ/AHQ) was about 8%, while the proportion of the wild type PrP genotype ARQ/ARQ was about 16% in the 2016 material [64]. Since there is over 70.000 sheep from different areas of Southern Norway in Nordfjella during summer we assume that the distribution of PrP genotypes in this area corresponds well with the general Norwegian sheep population. Thus, the investigated sheep from Nordfjella likely represent a variety of PrP genotypes, with unknown susceptibility for Norwegian reindeer CWD.

Choosing tissue type for screening purposes for a new, emerging prion disease is challenging, especially when the purpose is to investigate a possible interspecies transmission. Scrapie in sheep and CWD in cervids have similar pathogenesis with an early accumulation of prions in peripheral tissues and later involvement of the CNS [27, 31]. Based on this it was hypothesized that if sheep were able to acquire and propagate CWD prions, the pattern of dissemination would resemble that of scrapie and CWD, as well as BSE prions transmitted to sheep [23]. The early accumulation of prions in GALT, including the lymphoid follicle rich RAMALT [35] and the easy accessibility of the tissue, makes it ideal for sampling and screening purposes. Other lymphoid tissues such as the medial retropharyngeal lymph node, the third eyelid, distal jejunal lymph node, and the IPP have also proven to be tissues with high diagnostic value [32, 33, 65]. These tissues, however, were less accessible and difficult to sample in the abattoir setting. The IPP in sheep undergoes involution early in life [66], which makes it less reliable as sampling tissue once the animal gets older. Some studies suggest that accumulation of prions occurs earlier in the IPP than in other lymphoid organs [32], so we included IPP from 37 of the lambs in this study. Most of the IPP sections had a high number of follicles, showing that IPP is a suitable tissue for the screening of lambs. The number of RAMALT follicles also decreases with age, but the process is much slower than for the IPP [37]. This favors RAMALT as a more ideal tissue for screening different age groups.

The lymphoid aggregates in the rectum area are most abundant in the circumference starting from the mucocutaneous junction and approximately 1 cm proximally [35, 67]. Thus, sampling should target that region. We demonstrated that using the mucocutaneous junction as a guideline is beneficial to achieve RAMALT samples with a high number of lymphoid follicles important for prion disease diagnosis [27, 29, 30]. All RAMALT samples were additionally tested and confirmed negative with a commercial ELISA rapid test to increase confidence in the immunohistochemical results [68]. This commercial kit has been validated for use in lymphoid tissues of both sheep and cervids [69, 70]. Using ultra-sensitive methods such as RT-QuIC and PMCA could have increased the sensitivity of our study but were not used due to methodological constraints. One major difficulty for applying these tests in our study was the absence of standardized test reagents [71].

The social significance of the study lies in the guidance these results provide for management of CWD. This highlights that mitigation efforts directed against spread of the disease within reindeer populations are more important and urgent than efforts directed to prevent spread to sheep. We cannot, however, completely exclude the possibility for spread to sheep based on the results from the current study.

## Conclusion

Despite close interaction between reindeer and sheep in Nordfjella zone 1 prior to the culling of the reindeer population, we did not find any evidence of uptake and propagation of prions by sheep that have likely been exposed to environmental CWD prions. The absence of prions from sheep RAMALT and IPP may suggest that CWD prion uptake and propagation by sheep under the environmental conditions that exist in Nordfjella, is not prevalent. In addition, we observed that correctly sampled RAMALT tissue is easily accessible and yields a high mean number of lymphoid follicles when we use the recto-anal mucocutaneous junction as guideline for sampling. This makes the tissue an evident choice for mass sampling of lymphoid tissue.

## Data Availability

The datasets analyzed in the present study are available from the corresponding author upon request.

## Abbreviations

AEC: 3-Amino-9-ethylcarbazole
BSA: Bovine serum albumin
BSE: Bovine spongiform encephalopathy
CNS: Central nervous system
CWD: Chronic wasting disease
ELISA: Enzyme-linked immunosorbent assay
ENS: Enteric nervous system
GALT: Gut-associated lymphoid tissue
GPS: Global positioning system
HRP: Horseradish peroxidase
IHC: Immunohistochemistry
IPP: Ileal peyer’s patch
MCP: Minimum convex polygons
PMCA: Protein misfolding cyclic amplification
PrP: Prion protein
PrP^C^: Cellular prion protein
PrP^Sc^: Misfolded prion protein
RAMALT: Recto-anal mucosa associated lymphoid tissue
RT-QuIC: Real-time quacking induced conversion
SSE: Stratified squamous epithelium
UD: Utility distribution
